# Modeling Human Prostate Cancer Metastasis in Mice *via* Resection of Subcutaneous Allografts

**DOI:** 10.3389/fonc.2022.877536

**Published:** 2022-04-27

**Authors:** Lauren B. Peiffer, Jessica Hicks, Rebecca Y. Sosa, Angelo M. De Marzo, Karen S. Sfanos, Janielle P. Maynard

**Affiliations:** ^1^Department of Pathology, Johns Hopkins University School of Medicine, Baltimore, MD, United States; ^2^Department of Molecular and Comparative Pathobiology, Johns Hopkins University School of Medicine, Baltimore, MD, United States; ^3^Sidney Kimmel Comprehensive Cancer Center, Johns Hopkins University School of Medicine, Baltimore, MD, United States; ^4^Department of Urology, James Buchanan Brady Urological Institute, Johns Hopkins University School of Medicine, Baltimore, MD, United States

**Keywords:** prostate cancer_1_, metastasis_2_, Myc-CaP_3_, allograft_4_, mouse model_5_, resection_6_, FVB mouse_7_

## Abstract

The 5-year survival rate for patients diagnosed with distant metastatic prostate cancer in the United States is 30.6%. Therefore, there is a great need to develop *in vivo* model systems to study prostate cancer metastasis and to test potential therapeutics. Most murine prostate cancer metastatic models involve intracardiac or intraosseous implantation of cancer cells, which bypass the early stages of tumor cell migration and invasion. Herein we provide a detailed protocol for a novel method of resecting subcutaneous prostate cancer allografts in immunocompetent mice to produce spontaneous metastases and describe a pilot study using this method of tumor resection. Intact male FVB/NCrl mice (n = 9) were inoculated subcutaneously with Myc-CaP cells. Tumors were surgically resected, and mice were monitored for tumor recurrence. Animals were euthanized or died, and a full set of tissues was collected for histopathologic examination. Tumors took an average of 44 days (range 23–61) to reach 1.7 cm in any direction. All tumors were resectable, and resection of the tumors increased the study length by 70 days (range 30–121). One mouse was euthanized early of an unrelated cause, and of eight remaining mice, four developed tumor recurrence at the site of resection. One mouse developed bone metastases, one mouse developed metastases to the abdominal cavity, and two mice showed signs of local invasion. This study demonstrates that resection of subcutaneous Myc-CaP cell allografts in mice results in local tumor recurrence and the development of distant metastases, providing a new model system to study prostate cancer metastasis *in vivo*.

## 1 Introduction

The 5-year survival rate for patients with metastatic prostate cancer in the United States is 30.6% (1); therefore, there is a great need to develop *in vivo* model systems to study prostate cancer metastasis and to test potential therapeutics.

Subcutaneous tumor allografts offer a minimally invasive approach to *in vivo* prostate tumor modeling and allow the researcher to visually and quantitatively assess tumor volume over time. Myc-CaP cells are a common cell line used in prostate cancer allografts (2); however, primary Myc-CaP tumors grow rapidly and often reach allowed size limits prior to metastasizing. Therefore, most murine prostate cancer metastatic models involve intracardiac or intraosseous implantation of cancer cells (3). However, these methods bypass the early stages of tumor cell dissemination, migration, invasion, and intravasation.

Resection of primary tumors results in spontaneous metastases in mouse models of mammary and melanoma cancer (4–6). Therefore, we hypothesized that resecting subcutaneous prostate cancer allografts would result in spontaneous metastases. Herein we describe our protocol for subcutaneous Myc-CaP tumor inoculation and surgical resection. In a pilot study of nine mice, we report that surgical resection increased survival time by an average of 70 days and resulted in both abdominal and bone metastases. Possible applications for this method include 1) metastasis studies in immunocompetent, genetically unaltered mice; 2) studies to determine metastasis location, frequency, temporality, and patterns of invasion; 3) cell phenotyping studies that evaluate the expression of gene/proteins of interest in metastases versus primary tumor; 4) comparative studies of metastatic rates and location in genetically or pharmacologically altered tumor cells versus wild type; and 5) investigation of circulating tumor cells/cell-free DNA before versus after resection/surgical manipulation.

## 2 Materials and Equipment

### 2.1 Materials and Reagents

1. Institutional Animal Care and Use Committee (IACUC) approved the protocol for subcutaneous tumor inoculation and resection
2. Sterile gloves, arm covers, and mask
3. Sterile surgical space (biosafety hood or surgical suite)
4. Povidone-iodine/Betadine
5. Sterile drape
6. Alcohol
7. Matrigel (Corning 356234)
8. Myc-CaP cells (1 × 10^6^ per mouse)
9. Dulbecco’s modified Eagle’s medium (DMEM) containing l-glutamine
10. Heat-inactivated fetal bovine serum (FBS)
11. TrypLE Express Enzyme
12. Phosphate-buffered saline (PBS) without calcium and magnesium
13. Analgesia
14. Ophthalmic ointment
15. 1-ml syringe + 26G ½″ gauge needle (pre-chilled)
16. Hemocytometer, trypan blue
17. Hair removal cream
18. 70% ethanol
19. Isoflurane
20. Ice
21. Petri dishes
22. Disposable underpads

### 2.2 Equipment

1. Clippers
2. Cautery
3. Wound clips
4. Wound clip applicator
5. Wound clip remover
6. Heat pack
7. Institutional Animal Care and Use Committee (IACUC)-approved anesthesia equipment

## 3 Methods

### 3.1 Prepare Cell Suspensions

1. Culture Myc-CaP cells to 70%–80% confluency according to American Type Culture Collection (ATCC) protocols.
2. Wash with PBS, and dissociate cells using TrypLE express enzyme or 0.05% trypsin-EDTA at 37°C for 3–6 min. Neutralize TrypLE with an equivalent volume of DMEM containing l-glutamine and with 10% FBS (complete media).
3. Collect cell suspension in a conical tube and spin at 300 × *g* for 5 min. Discard TrypLE/media and resuspend pellet in PBS. Spin at 300 × *g* for 5 min. Re-suspend in 2 ml of PBS.
4. Count cells and dilute cells to 20 × 10^6^ cells/ml in PBS. Chill on ice. With the use of pre-chilled pipette tips, add equal parts of the cell suspension to Matrigel to form 1 × 10^6^ cells per 100 µl of suspension. *Note: Thaw Matrigel on ice (may be done overnight at 4°C on ice). Matrigel will form a gel above 10°C.*
5. Aspirate 100 µl of Matrigel–cell suspension in each pre-chilled syringe. Keep on ice until inoculation.

### 3.2 Tumor Inoculation

1. Anesthetize the animals according to IACUC-approved protocol.
2. Apply ophthalmic ointment to eyes to prevent drying.
3. Remove fur at and around the site of inoculation in an area separate from surgical setup:
  a. Shave approximately 1.5 × 1.5 patch of fur over the right flank.
  b. Apply a thin layer of hair removal cream with sterile gauze or cotton-tipped applicator and let sit for ~5 to 10 s.
  c. Immediately wipe with sterile water-soaked gauze to remove remaining hair.
4. Disinfect the surgical site with 2-min total contact time of povidone-iodine solution followed by 70% ethanol.
5. Tent skin away from the body and insert the needle through the skin into the subcutaneous pocket.
6. Inject 100 µl of cell suspension (~1 million cells) very slowly.
7. Remove needle and release skin.
8. Recover mouse from anesthesia.
9. Return the mouse to the home cage and monitor for signs of distress.

### 3.3 Monitor Tumor Growth

1. Check animals routinely based on IACUC-approved protocol.
2. Tumors are generally palpable by 10 days post-inoculation. Measure tumor length and width using digital calipers, every 1 to 3 days.
3. Estimate tumor volume with the following equation: (length × width^2^)/2.
4. Perform surgical resection once the tumor approaches allowable size limits.

### 3.4 Surgical Resection

1. Anesthetize the animals, apply ophthalmic ointment, remove fur, and disinfect the surgical site as outlined in Section 3.2.
2. Administer analgesia according to IACUC-approved protocol.
3. Apply sterile drape.
4. Incise curvilinear incision along the base of the tumor caudally to cranially using tissue-separator scissors.
5. Blunt dissect out the tumor, separating it from the skin when possible:
  a. If adhered to the skin, remove only what skin is necessary.
  b. Cauterize blood vessels as necessary.
6. Place tumor in PBS until finished with surgery.
7. Check for bleeding, and ensure all blood vessels are cauterized.
8. Close surgical incision with wound clips or subcuticular, simple interrupted sutures.
9. Allow the mice to recover from anesthesia and monitor until fully awake.
10. Return the mouse to the home cage.
11. Manipulate or store tumor tissue as needed:
  a. For example, transect the tumor using a scalpel or sterile razor blade, fix one portion of the tumor in 10% neutral buffered formalin (10× volume formalin to the volume of tumor), and flash freeze the other portion in liquid nitrogen.

### 3.5 Monitor Tumor Growth

1. Monitor tumor growth as outlined in Section 3.3.
2. Monitor for the following based on our experience:
  a. Tumor recurrence at the site of primary tumor
  b. Tumor ulceration
  c. Hematoma formation at the site of primary tumor
  d. Lameness and leg swelling (in the event of bone metastasis)
  e. Abdominal swelling (in the event of abdominal cavity metastases)
  f. Rapidly increased body weight
  g. General signs of malaise: ruffled fur, hunched posture, and decreased activity

### 3.6 RNA *In Situ* Hybridization and Immunohistochemistry

RNA *in situ* hybridization (RISH) was performed using the RNAscope 2.5 FFPE Brown Reagent Kit (Cat. No. 322310, Advanced Cell Diagnostics, Newark, CA, USA) following the manufacturer’s instructions. In brief, formalin-fixed paraffin-embedded (FFPE) tissue sections on glass slides were baked at 60°C for 30 min followed by deparaffinization in three changes of 100% xylene for 10 min each and two changes of 100% ethanol for 1 min each. Next, the slides were treated with hydrogen peroxide for 10 min at room temperature to block endogenous peroxidases. The slides were then placed in boiling buffer for 15 min in a steamer before being treated with protease digestion buffer for 30 min at 40°C. The slides were hybridized with a custom RNAscope probe designed against either human *MYC* mRNA (probe region 536–1995, NCBI seq. #NM_002467.4) that we have found to not cross-hybridize to mouse *Myc* mRNA (7), or mouse *Ppib* (probe region 98-856, NCBI seq. #NM_011149.2) also known as cyclophilin B as a positive control mRNA for 2 h at 40°C, followed by signal amplification. The color reaction used 3,3′-diaminobenzidine (DAB) for 10 min at room temperature. Sections were counterstained with Gill’s hematoxylin. All FFPE blocks and sections on slides were maintained at −20°C soon after collection to reduce the loss of signals (7). Slides were scanned with a whole tissue slide scanner. Slides were viewed, and images were captured using Concentriq (Proscia Inc., Philadelphia, PA, USA).

### 3.7 Immunohistochemistry

H&E staining was completed by the Johns Hopkins University Oncology Tissue Services Core. Immunohistochemistry (IHC) was performed using a Ventana DISCOVERY ULTRA Autostainer using the DISCOVERY anti-HQ HRP kit as previously described (8). We validated an automated assay against androgen receptor (AR) using a monoclonal antibody (Cat. No. 5153, Cell Signaling, Danvers, MA, USA) at 1:200 dilution using AR-positive LNCaP cells and AR-negative PC3 cells. Slides were scanned and images processed as described above.

## 4 Results

**Figure 1:**
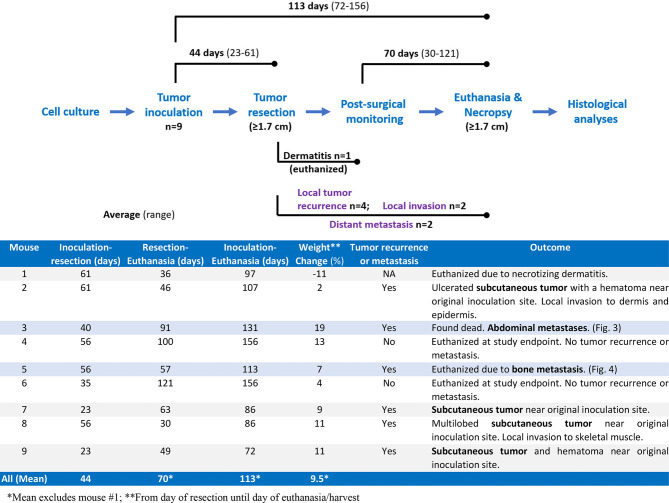
Summary of pilot study. Nine mice were inoculated with Myc-CaP cells. Tumors were resected from all nine mice once tumor size reached 1.7 cm in any direction, an average of 44 days (range 23–61 days) after engraftment. One mouse was euthanized due to dermatitis of an unknown cause. Four of the remaining eight mice had recurrent tumors of which two showed signs of local invasion. Two of the remaining four mice had distant metastasis. Seven mice were euthanized on average 70 days (range 30–121 days) post-resection when recurrent tumors reached 1.7 cm, tumors became ulcerated, or mice were moribund. One mouse was found dead.

**Figure 2:**
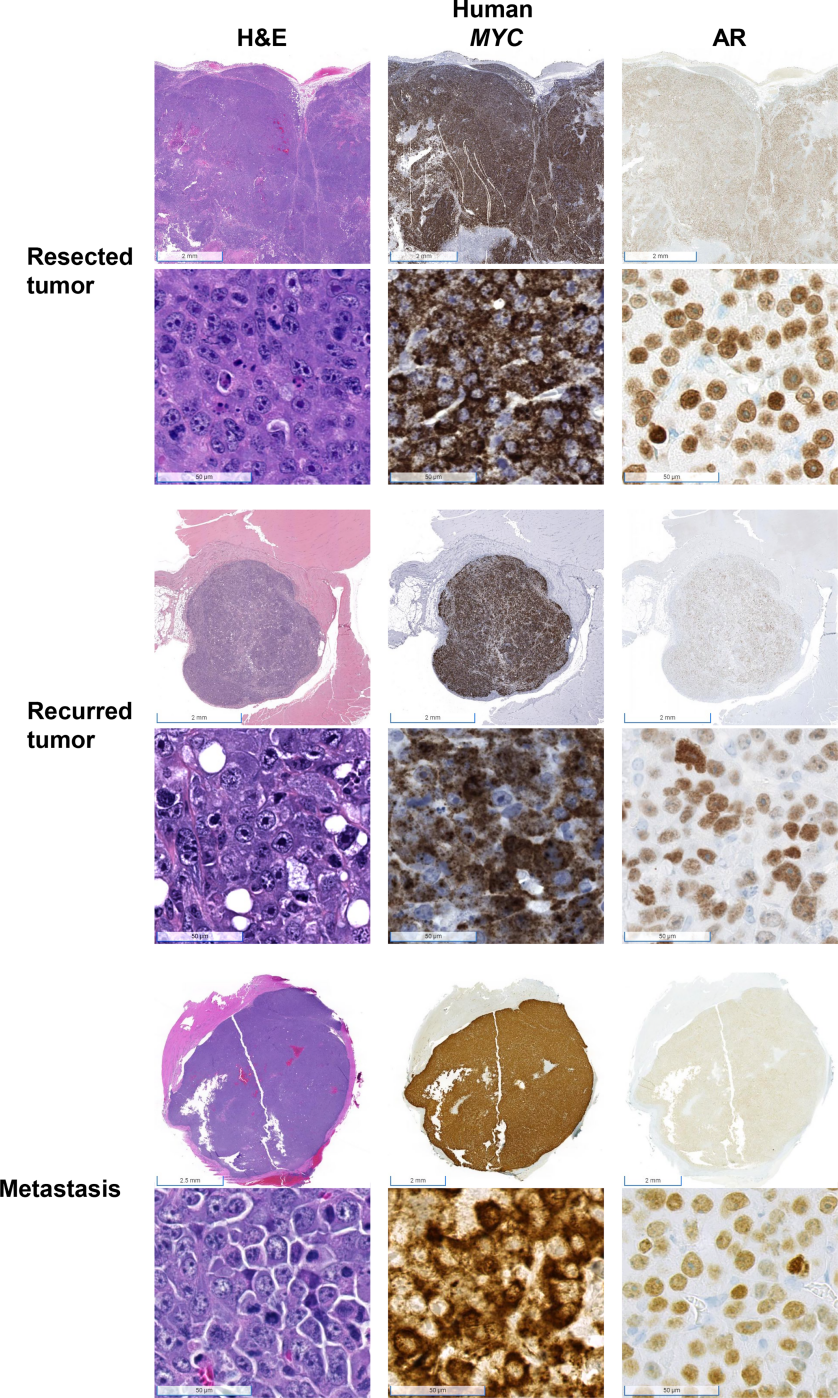
Confirmation that tumors were derived from Myc-CaP cells. H&E staining of tumor cells from resected, recurred, and metastatic tumors demonstrate abundant cytoplasmic expression of human *MYC* and nuclear AR protein expression, consistent with Myc-CaP cells. Scale bar = 2 mm (top), 50 µm (bottom).

We performed Myc-CaP tumor inoculation and surgical resection on nine intact male FVB/nCrl mice. The results of this pilot study are summarized in **Figure 1**. Tumors took an average of 44 days (range 23–61) to reach 1.7 cm in any direction. All tumors were resectable, and mice survived an average of 70 days (range 30–121) after surgical resection. The total average timespan from tumor inoculation to euthanasia or death was 113 days (range 72–156). Mouse #1 was euthanized early due to necrotizing dermatitis of an unknown cause (**Figure 1**). Of the eight remaining mice, four developed tumor recurrence at or near the site of resection. We confirmed that resected and recurred tumors were Myc-CaP cells by demonstrating abundant cytoplasmic expression of human *MYC* mRNA by RISH and nuclear AR expression by IHC (**Figure 2**). Two of the four mice in which tumor recurred exhibited signs of local invasion. Of the four remaining mice, two mice had distant metastases.

**Figure 3:**
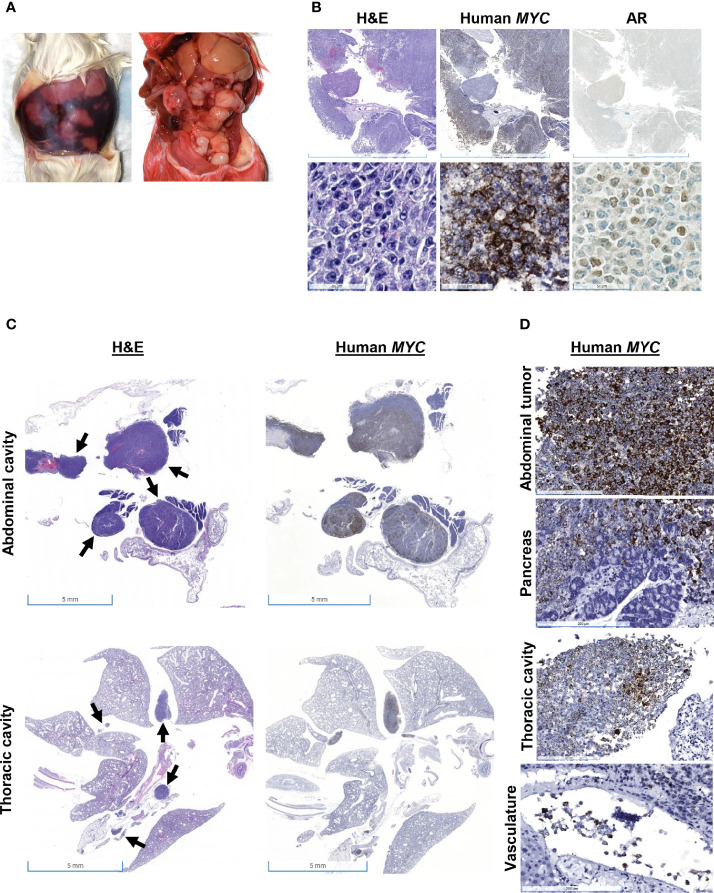
Gross and histologic findings for Mouse #3. This animal was found dead with **(A)** 3 ml of serosanguinous fluid and approximately 20 tan nodules up to 1 cm in diameter in the abdominal cavity, which expresses **(B)** both human *MYC* mRNA and AR protein expression. Scale bar = 5 mm (top), 50 µm (bottom). **(C)** H&E showed neoplastic cells present within the thoracic and abdominal cavities, and human *MYC* expression confirmed that these tumors were derived from Myc-CaP cells (scale bar = 5 mm). Arrows show neoplastic cells. **(D)** Human *MYC*-positive cells invaded the pancreas and were present intravascularly. Scale bar = 200 µm.

Mouse #3 was found dead with approximately 3 ml of serosanguinous fluid and approximately 20 tan nodules up to 1 cm in diameter in the abdominal cavity (**Figure 3A**). There was no apparent tumor recurrence at or near the site of inoculation. There was a 19% increase in body weight post-resection (**Figure 1**). Histologically, nodules were composed of cells positive for human *MYC* mRNA and AR (**Figure 3B**). H&E showed neoplastic cells present within the mediastinum, thoracic cavity, abdominal fat, and mesentery and the surrounding intestines (**Figure 3C**). Human *MYC*-positive cells were observed in the pancreas and intravascularly (**Figure 3D**). Mesenteric lymph nodes were not visualized in sections of the mesentery and pancreas, which is extremely unusual and suggests that lymph nodes may have been effaced and replaced by metastatic cells.

**Figure 4:**
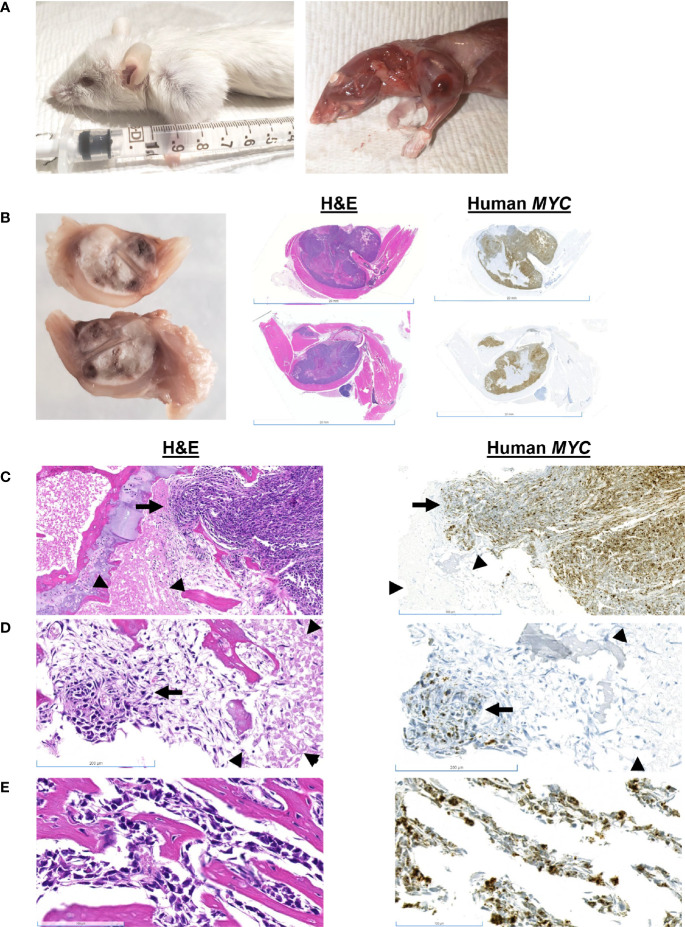
Gross and histologic findings for Mouse #5. **(A)** This animal presented clinically with a hard swelling of the left front leg. **(B)** The swelling was due to a well-demarcated, 1 cm, white mass that involved the humerus. Scale bar = 20 mm. **(C)** Sheets of neoplastic cells surround the bone; they multifocally disrupt the cortex (arrow). Scale bar = 500 µm. The medullary cavity is occupied by necrotic cells (arrowheads) and **(D)** nests of neoplastic cells (arrow). Scale bar = 200 µm. **(E)** Sub-periosteally, bone trabeculae radiate from the cortex and are surrounded by neoplastic cells. Scale bar = 100 µm.

Mouse #5 was euthanized due to lameness and hard swelling of the left front leg (**Figure 4A**). There was no apparent tumor recurrence at or near the site of inoculation. There was a 7% increase in body weight post-resection (**Figure 1**). At necropsy, the swelling was due to a well-demarcated, 1 cm, white mass that involved the humerus. Histologically, the mass consisted of sheets of *MYC*-positive neoplastic cells surrounding the humerus (**Figure 4B**). Neoplastic cells multifocally disrupted the bone cortex (**Figure 4C**). Cells within the medullary cavity were mostly necrotic (**Figures 4C, D**); however, there were multifocal nests of *MYC*-positive neoplastic cells within the medullary cavity (**Figure 4D**). Sub-periosteal cortical bone was multifocally proliferative, with bone trabeculae radiating from the cortex (**Figure 4E**).

**Figure 5:**
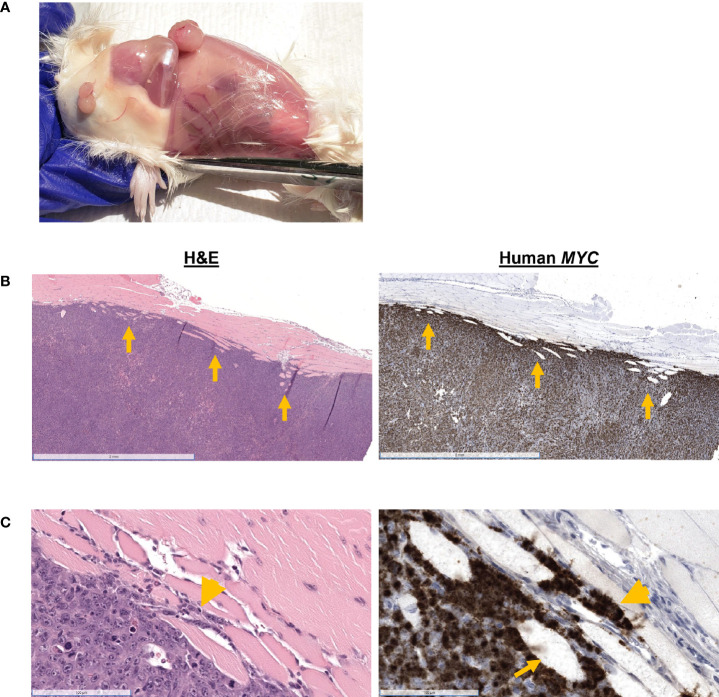
Gross and histologic findings for Mouse #8. This mouse was euthanized when the recurrent tumor measured 1.7 cm in size. **(A)** Subcutaneous nodules were located in the pelvic region, over the right thigh. **(B)** H&E showed skeletal muscle invasion (arrows) of *MYC*-positive neoplastic cells. Scale bar = 2 mm. **(C)** *MYC*-positive cells are observed between myofibers (arrowhead) and completely entrapping myofibers (arrow). Scale bar = 100 µm.

Mouse #8 was euthanized when the recurred subcutaneous tumor exceeded 1.7 cm in one direction. Multiple masses were grossly visible, and the mouse gained 11% body weight post-resection (**Figure 1**). At necropsy, four nodules were observed: at the pelvic region, two over the right thigh, and one at the site of inoculation (**Figure 5A**). H&E staining of the mass over the thigh showed local skeletal muscle invasion of neoplastic cells (**Figures 5B, C**). *MYC*-positive cells are observed between myofibers at the periphery of the tumor (**Figure 5C**), and there were myofibers that were completed surrounded by *MYC*-positive cells (**Figures 5B, C**). H&E of an ulcerated subcutaneous tumor with a hematoma located on the right hind leg near the original inoculation site of Mouse #2 also showed neoplastic cells invading the dermis and epidermis (**Figure 1**).

## 5 Discussion

Herein we describe a protocol for surgical resection of subcutaneous Myc-CaP allograft tumors. We performed this protocol on nine mice and show that two mice developed distant metastases and four mice had tumor recurrence, two of which had signs of local invasion. We show that by resecting tumors, we increase the time to euthanasia or death by an average of 70 days.

There are limited reports of metastases in murine prostate cancer allograft models. One group showed that orthotopic allografted Myc-CaP cells metastasized to abdominal lymph nodes, liver, and lungs (9, 10). Another study demonstrated that B6CaP, a serially transplantable allograft line generated from Hi-Myc mouse lymph node metastasis, results in metastasis when allografted subcutaneously or orthotopically into C57BL/6J mice (11). We offer an additional approach that utilizes the convenience and efficiency of subcutaneous allografting as well as the use of an established prostate cancer cell line that may be cultured using standard cell culture protocols. The protocol we describe may be manipulated to augment its application in modeling prostate cancer metastasis. For instance, Myc-CaP cells transduced with green fluorescent protein (GFP) may be monitored by *in vivo* fluorescence imaging to track the dissemination and invasion of prostate cancer cells during metastasis.

Our pilot study is limited by the number of animals used. Further, this protocol requires an intermediate level of surgical expertise. Without *in vivo* imaging capabilities, thorough necropsies and extensive histopathologic examinations are necessary to assess metastases. Even with these limitations, the described protocol models spontaneous prostate cancer metastasis demonstrating a useful experimental option for prostate cancer research.

## Data Availability Statement

The original contributions presented in the study are included in the article/supplementary material. Further inquiries can be directed to the corresponding author.

## Ethics Statement

The animal study was reviewed and approved by Johns Hopkins University Animal Care and Use Committee.

## Author Contributions

LP and JM contributed to the conceptualization of the study, analyzed data, and were involved in manuscript writing. LP, RS, and JM performed experiments. LP and AD are pathologists that characterized mouse tissues. AD and KS provided supervision and resources. All authors were involved in manuscript editing.

## Funding

This study was funded by Prostate Cancer Foundation Challenge Award 19CHAS03.

## Conflict of Interest

The authors declare that the research was conducted in the absence of any commercial or financial relationships that could be construed as a potential conflict of interest.

## Publisher’s Note

All claims expressed in this article are solely those of the authors and do not necessarily represent those of their affiliated organizations, or those of the publisher, the editors and the reviewers. Any product that may be evaluated in this article, or claim that may be made by its manufacturer, is not guaranteed or endorsed by the publisher.
